# Adverse Effects from Clenbuterol and Ractopamine on Nematode *Caenorhabditis elegans* and the Underlying Mechanism

**DOI:** 10.1371/journal.pone.0085482

**Published:** 2014-01-21

**Authors:** Ziheng Zhuang, Yunli Zhao, Qiuli Wu, Min Li, Haicui Liu, Lingmei Sun, Wei Gao, Dayong Wang

**Affiliations:** 1 School of Pharmaceutical Engineering and Life Sciences, Changzhou University, Changzhou, China; 2 Key Laboratory of Environmental Medicine Engineering in Ministry of Education, Medical School of Southeast University, Nanjing, China; 3 Jiangsu Province Product Quality Supervision and Inspection Institute, Nanjing, China; 4 Xiuli Biological Technology Co., Ltd. Changzhou, China; Katholieke Universiteit Leuven, Belgium

## Abstract

In the present study, we used *Caenorhabditis elegans* assay system to investigate *in vivo* toxicity from clentuberol and ractopamine and the possible underlying mechanism. Both acute and prolonged exposures to clentuberol or ractopamine decreased brood size and locomotion behavior, and induced intestinal autofluorescence and reactive oxygen species (ROS) production. Although acute exposure to the examined concentrations of clentuberol or ractopamine did not induce lethality, prolonged exposure to 10 µg/L of clentuberol and ractopamine reduced lifespan. At relatively high concentrations, ractopamine exhibited more severe toxicity than clentuberol on nematodes. Overexpression of *sod-2* gene encoding a Mn-SOD to prevent induction of oxidative stress effectively inhibited toxicity from clentuberol or ractopamine. Besides oxidative stress, we found that clentuberol might reduce lifespan through influencing insulin/IGF signaling pathway; however, ractopamine might reduce lifespan through affecting both insulin/IGF signaling pathway and TOR signaling pathway. Ractopamine more severely decreased expression levels of *daf-16*, *sgk-1*, *skn-1*, and *aak-2* genes than clentuberol, and increased expression levels of *daf-2* and *age-1* genes at the examined concentration. Therefore, the *C. elegans* assay system may be useful for assessing the possible toxicity from weight loss agents, and clentuberol and ractopamine may induce toxicity through different molecular mechanisms.

## Introduction

Illegal or unsuitable use of weigh loss agents has gradually become a public health concern [1]–[2]. Clenbuterol, a typical weight loss agent, is a kind of β2-adrenergic agonist, and was illegally used as a feed additive to improve production performance and a carcass composition in many countries [3]–[4]. With the increased use of internet sales, the internet has made this even banned product to be readily accessible for the aim of weight loss or dieting addition [2]. Ractopamine, a synthetic β2-adrenoceptor agonist, is now widely used as a feed additive in the United States to promote a reduction in body fat and to enhance muscle growth in cattle, pigs, or turkeys [5].

Among the used weight loss agents, clenbuterol and ractopamine belong to products that may have health hazards upon accidental or intentional exposure and ingestion [2], [6]. The *in vitro* study has indicated that clenbuterol exhibited potential toxicity on structure and function of trypsin, an important digestive enzyme, and stimulated guinea-pig heart rate [4], [7]. Following consumption of meat or liver from clenbuterol administrated cattle, intoxication cases were described [7]. Moreover, at least the toxic effects on cardiovascular systems (such as tachycardia and hypertension) are considered to be of clinical relevance [7]–[9]. More recently, it was further reported that ractopamine administration might cause the myocardial toxicity in dogs [5].

Several toxicological studies have been performed for clenbuterol. Administration of growth-promoting doses of clenbuterol adversely affected the liver function in female pigs [10]. Dietary administration of clenbuterol decreased androgen receptor (AnR) expression in testicle, glucocorticoid receptor (GR) expression in lymphoid tissues, and β-adrenergic receptor (β-AR) expression in targeted organs of chickens [3]. Clenbuterol caused an impairment of collagen turnover by down-regulating MMP-9 activity [11]. Clenbuterol not only enhanced muscle fiber size but also increased expression of GATA-2 protein in skeletal muscle of rat uterus [12]. The preferential involvement of calpain 2 autolysis was found for clenbuterol-induced skeletal muscle remodelling in rats [13]. Overexpression of calpastatin in skeletal muscle of mice prevented clenbuterol-induced muscle hypertrophy and phenotypic shift [14]. However, so far no toxicological study on ractopamine has been performed.

Nematode *Caenorhabditis elegans*, an important model animal used in various fields, has invariant and fully described developmental program, well-characterized genome, short and prolific life cycle, and small body size [15]–[16]. The success of *C. elegans* as a model animal has attracted the increased attention in the fields of both biomedical science and toxicology [16]–[18]. *C. elegans* has been widely accepted and utilized as an important alternative animal model for toxicity testing [16], [19]–[20]. A number of toxicity studies have been conducted with the aid of both lethal and sub-lethal endpoints for metallic salts [21]–[31], organic compounds [32]–[36], drugs [37]–[40], and engineered nanomaterials [41]–[50]. *C. elegans* is useful for toxicological studies from whole-animal level down to single cell level [51]. A series of studies have found that toxicity for toxicants in *C. elegans* is similar to that observed in mammals [16], [19], implying that the toxicological studies performed in *C. elegans* will closely reflect the effects to be observed in mammalian models for most compounds tested.

In the present study, we first compared the toxicity between clentuberol and ractopamine with the aid of a series of endpoints in *C. elegans*. Moreover, considering the fact that we still know limited information about toxicological mechanism for the clentuberol and especially the ractopamine, we examined the underlying mechanism for toxicity from clentuberol and ractopamine. Our results here will be useful for the further understanding multiple toxicities from clentuberol and ractopamine and the underlying mechanism.

## Results

### Comparison of lethality and growth in clentuberol or ractopamine exposed nematodes

**Figure 1:**
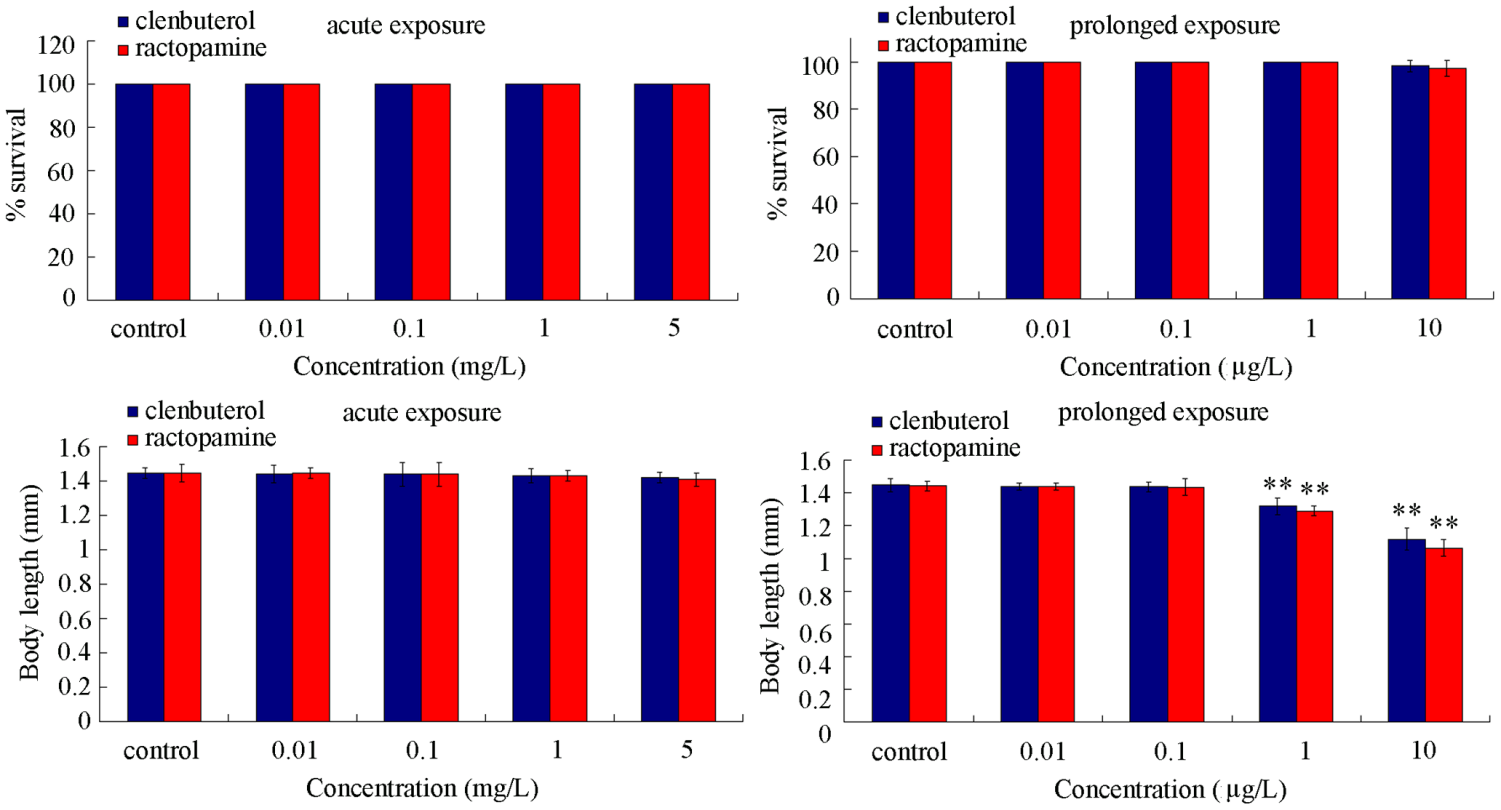
Comparison of lethality and growth in nematodes exposed to different concentrations of clenbuterol or ractopamine. Exposures were performed from the young adult for 24-hr (acute exposure) or from L1-larvae to adult (prolonged exposure). Fifty nematodes were examined per treatment for lethality assay, and twenty nematodes were examined per treatment for growth assay. Bars represent mean ± S.E.M. **P<0.01.

Considering the fact that many toxicants at the low concentrations may have the adverse effects on nematodes after prolonged exposure [18], [25], [44]–[49], we performed both the acute exposure and the prolonged exposure for clentuberol or ractopamine. Concentrations of 0.01–5 mg/L were used for acute exposure to clentuberol or ractopamine. After acute exposure from young adult stage for 24-hr, both clentuberol and ractopamine did not induce lethality and alteration of body length in nematodes (Fig. 1). Concentrations of 0.01–10 µg/L were used for prolonged exposure to clentuberol or ractopamine. After prolonged exposure from L1-larvae to the adult stage, although both clentuberol and ractopamine still did not induce lethality of nematodes, both clentuberol and ractopamine at concentrations more than 1 µg/L significantly reduced body length of nematodes (Fig. 1).

### Comparison of reproduction and locomotion behavior in clentuberol or ractopamine exposed nematodes

**Figure 2:**
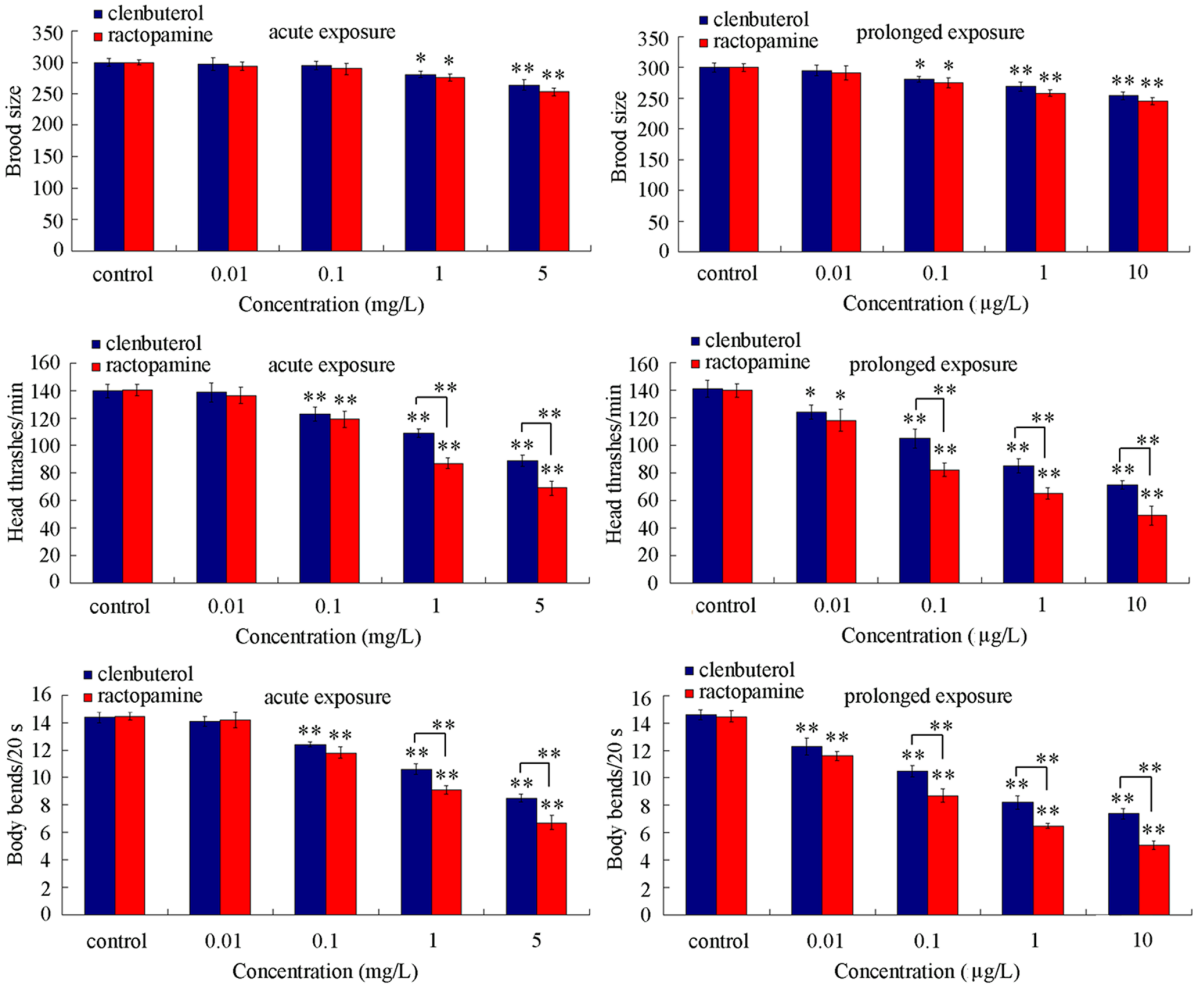
Comparison of brood size and locomotion behavior in nematodes exposed to different concentrations of clenbuterol or ractopamine. Locomotion behavior of nematodes was evaluated by endpoints of head thrash and body bend. Exposures were performed from the young adult for 24-hr (acute exposure) or from L1-larvae to adult (prolonged exposure). Twenty nematodes were examined per treatment for brood size assay, and fifty nematodes were examined per treatment for locomotion behavior assay. Bars represent mean ± S.E.M. *P<0.05, **P<0.01.

Reproductive organ and neuron may be important secondary targeted organs for toxicants in nematodes [18], [44], [48]–[49], [52]. Acute exposure to clenbuterol or ractopamine at concentrations of 0.01–0.1 mg/L and prolonged exposure to 0.01 µg/L of clenbuterol or ractopamine did not significantly alter brood size (Fig. 2). Acute exposure to 0.01 mg/L of clenbuterol or ractopamine did not significantly influence locomotion behavior of nematodes (Fig. 2). In contrast, acute exposure to clenbuterol or ractopamine at concentrations more than 1 mg/L and prolonged exposure to clenbuterol or ractopamine at concentrations more than 0.1 µg/L significantly reduced brood size (Fig. 2). Acute exposure to clenbuterol or ractopamine at concentrations more than 0.1 mg/L and prolonged exposure to clenbuterol or ractopamine at concentrations more than 0.01 µg/L significantly decreased locomotion behavior of nematodes (Fig. 2). More interestingly, we observed that acute exposure to 1–5 mg/L of ractopamine and prolonged exposure to 0.1–10 µg/L of ractopamine exhibited more severe toxicity on locomotion behavior than clenbuterol in nematodes, although ractopamine at the examined concentrations still showed the similar toxicity on brood size to clenbuterol in nematodes (Fig. 2).

### Comparison of intestinal autofluorescence in clentuberol or ractopamine exposed nematodes

**Figure 3:**
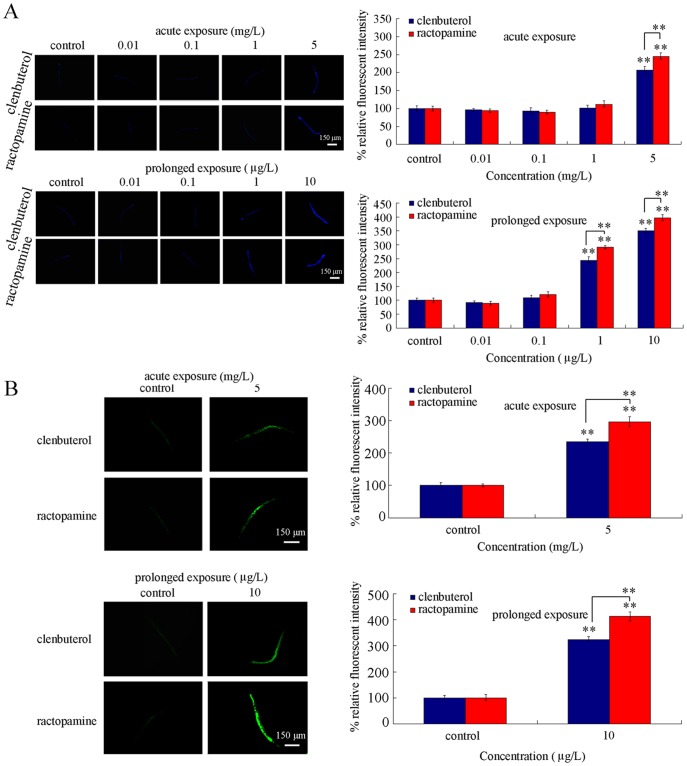
Comparison of intestinal autofluorescence (A) and ROS production (B) in nematodes exposed to clenbuterol or ractopamine. Exposures were performed from the young adult for 24-hr (acute exposure) or from L1-larvae to adult (prolonged exposure). Twenty nematodes were examined per treatment. Bars represent mean ± S.E.M. **P<0.01.

Intestine is the primary targeted organ for toxicants in nematodes [18], [41], [43], [48]. Acute exposure to clenbuterol or ractopamine at concentrations of 0.01–0.1 mg/L and prolonged exposure to clenbuterol or ractopamine at concentrations of 0.01–0.1 µg/L did not induce the significant intestinal autofluorescence compared with control (Fig. 3A). In contrast, acute exposure to 5 mg/L of clenbuterol or ractopamine and prolonged exposure to clenbuterol or ractopamine at concentrations of 1–10 µg/L significantly induced the intestinal autofluorescence compared with control (Fig. 3A). More interestingly, we observed that acute exposure to 5 mg/L of ractopamine and prolonged exposure to 1–10 µg/L of ractopamine induced more pronounced intestinal autofluorescence than clenbuterol in nematodes (Fig. 3A).

### Comparison of intestinal reactive oxygen species (ROS) production in clentuberol or ractopamine exposed nematodes

In *C. elegans*, toxicants usually cause damage on animals by inducing oxidative stress [18], [29], [53]. We further examined the intestinal ROS production in clentuberol or ractopamine exposed nematodes. After acute exposure, both clentuberol and ractopamine at the concentration of 5 mg/L induced the significant intestinal ROS production, and acute exposure to ractopamine at the concentration of 5 mg/L resulted in more severe intestinal ROS production than clentuberol (Fig. 3B). After prolonged exposure, similarly, both clentuberol and ractopamine at the concentration of 10 µg/L caused significant intestinal ROS production, and prolonged exposure to ractopamine at the concentration of 10 µg/L induced more severe intestinal ROS production than clentuberol (Fig. 3B). These data suggest that toxicity from clentuberol or ractopamine may be closely associated with the induction of oxidative stress in nematodes.

### Comparison of lifespan in clentuberol or ractopamine exposed nematodes

**Figure 4:**
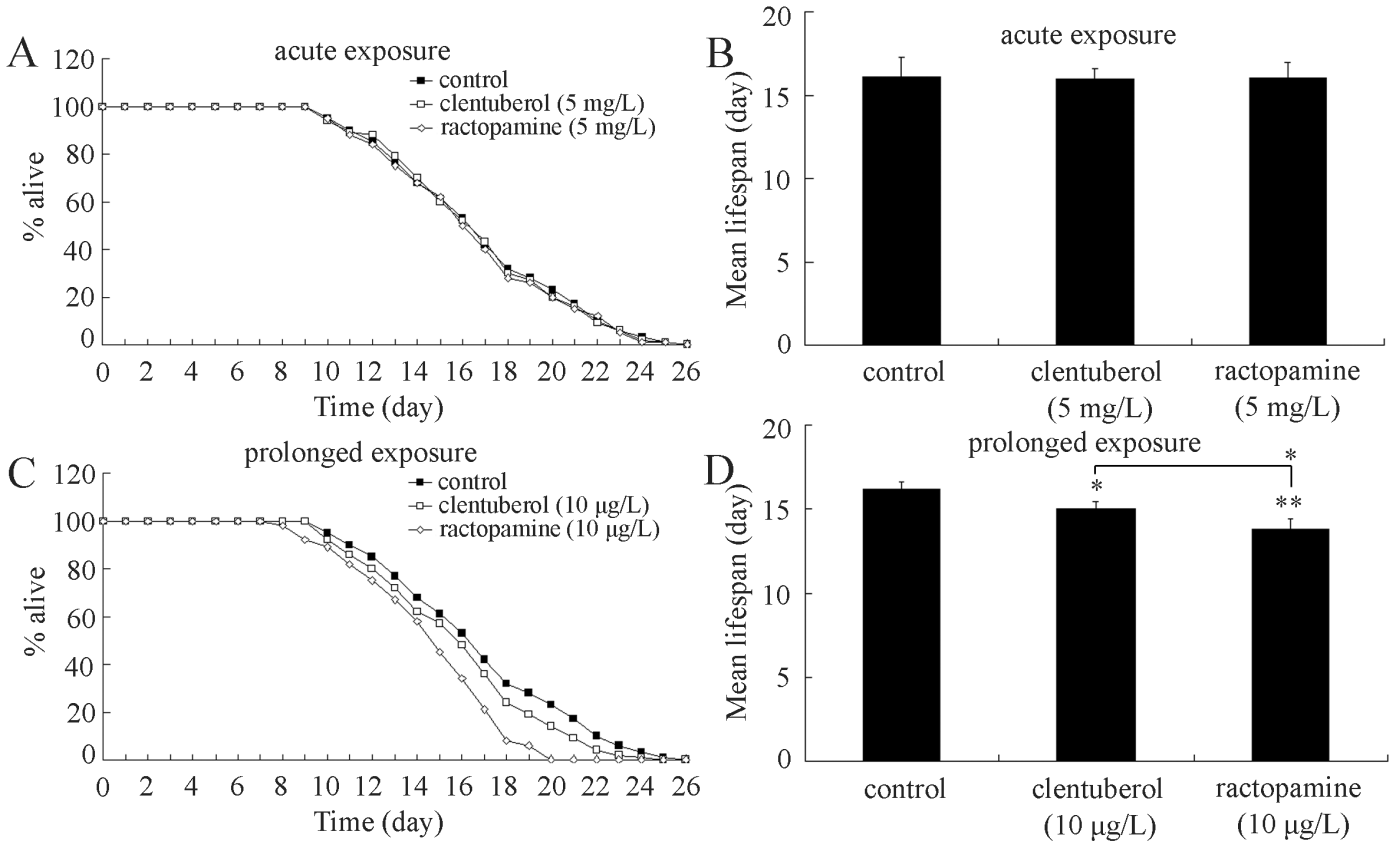
Comparison of lifespan in nematodes exposed to clenbuterol or ractopamine. (A and C) Lifespan curves of nematodes exposed to clenbuterol or ractopamine. (B and D) Comparison of mean lifespans in nematodes exposed to clenbuterol or ractopamine. Exposures were performed from the young adult for 24-hr (acute exposure) or from L1-larvae to adult (prolonged exposure). Thirty nematodes were examined per treatment. Bars represent mean ± S.E.M. *P<0.05, **P<0.01.

We further investigated the effects of exposure to clentuberol and ractopamine on lifespan in nematodes. Lifespan is an important endpoint and may reflect the long-term effects of a specific toxicant in nematodes [52], [54]. Acute exposure to clentuberol or ractopamine at the concentration of 5 mg/L did not significantly alter lifespan of nematodes (Figs. 4A and 4B). In contrast, prolonged exposure to clentuberol or ractopamine at the concentration of 10 µg/L significantly reduced the lifespan of nematodes (Figs. 4C and 4D). Moreover, we found that prolonged exposure to ractopamine at the concentration of 10 µg/L more severely inhibited the lifespan of nematodes than clentuberol (Figs. 4C and 4D).

### Overexpression of SOD-2 prevented the toxicity of clentuberol or ractopamine on nematodes

**Figure 5:**
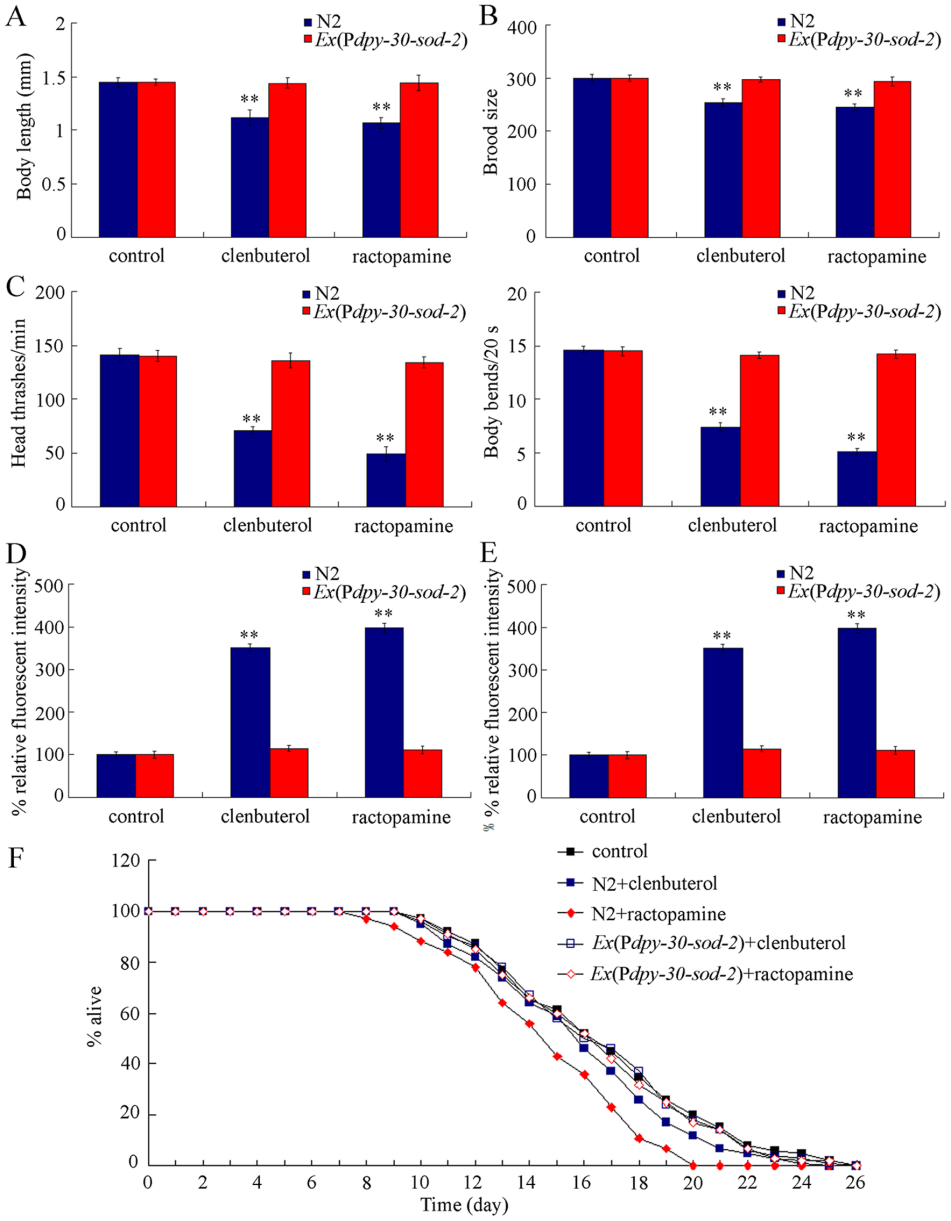
Effects of overexpression of *sod-2* gene on toxicity from clentuberol or ractopamine in *C. elegans*. (A) Effects of overexpression of *sod-2* gene on growth in clentuberol or ractopamine exposed nematodes. Twenty nematodes were examined per treatment. (B) Effects of overexpression of *sod-2* gene on brood size in clentuberol or ractopamine exposed nematodes. Twenty nematodes were examined per treatment. (C) Effects of overexpression of *sod-2* gene on locomotion behavior in clentuberol or ractopamine exposed nematodes. Fifty nematodes were examined per treatment. (D) Effects of overexpression of *sod-2* gene on intestinal autofluorescence in clentuberol or ractopamine exposed nematodes. Twenty nematodes were examined per treatment. (E) Effects of overexpression of *sod-2* gene on intestinal ROS production in clentuberol or ractopamine exposed nematodes. Twenty nematodes were examined per treatment. (F) Effects of overexpression of *sod-2* gene on lifespan in clentuberol or ractopamine exposed nematodes. Thirty nematodes were examined per treatment. Exposures were performed from L1-larvae to adult (prolonged exposure) at the concentration of 10 µg/L. Bars represent mean ± S.E.M. **P<0.01.

In *C. elegans*, *sod-2* and *sod-3* genes encode Mn-SODs, which function in protecting animals from the damage from oxidative stress [45], [55]. Previous studies have demonstrated that overexpression of Mn-SODs inhibited the oxidative stress and prevented the toxicity from metals and nanomaterials [45], [55]. To examine the role of oxidative stress in inducing the toxicity from clentuberol or ractopamine on nematodes, we investigated the effects of overexpression of *sod-2* gene in all cells of nematodes (*Ex*(P*dpy-30-sod-2*)) on toxicity from clentuberol or ractopamine. Transgenic strain of *Ex*(P*dpy-30-sod-2*) had no deficits in body length, brood size, head thrash, and body bend, and did not have significant intestinal autofluorescence and intestinal ROS production (Figs. 5A–5E). After prolonged exposure to 10 µg/L of clentuberol or ractopamine, we did not detect the significant decreases in body length, brood size, head thrash, and body bend in nematodes overexpressing *sod-2* gene compared with control (Figs. 5A–5C). Moreover, after prolonged exposure to 10 µg/L of clentuberol or ractopamine, we did not observe the significant induction of both intestinal autofluorescence and intestinal ROS production in nematodes overexpressing the *sod-2* gene compared with control (Figs. 5D and 5E). Nematodes overexpressing *sod-2* gene had the similar lifespan to that of wild-type N2 (data not shown). After prolonged exposure to 10 µg/L of clentuberol or ractopamine, our results further demonstrate that no significant reduction of lifespan was observed in nematodes overexpressing *sod-2* gene compared with control (Fig. 5F).

### Molecular mechanism for clentuberol and ractopamine to reduce the lifespan of nematodes

**Figure 6:**
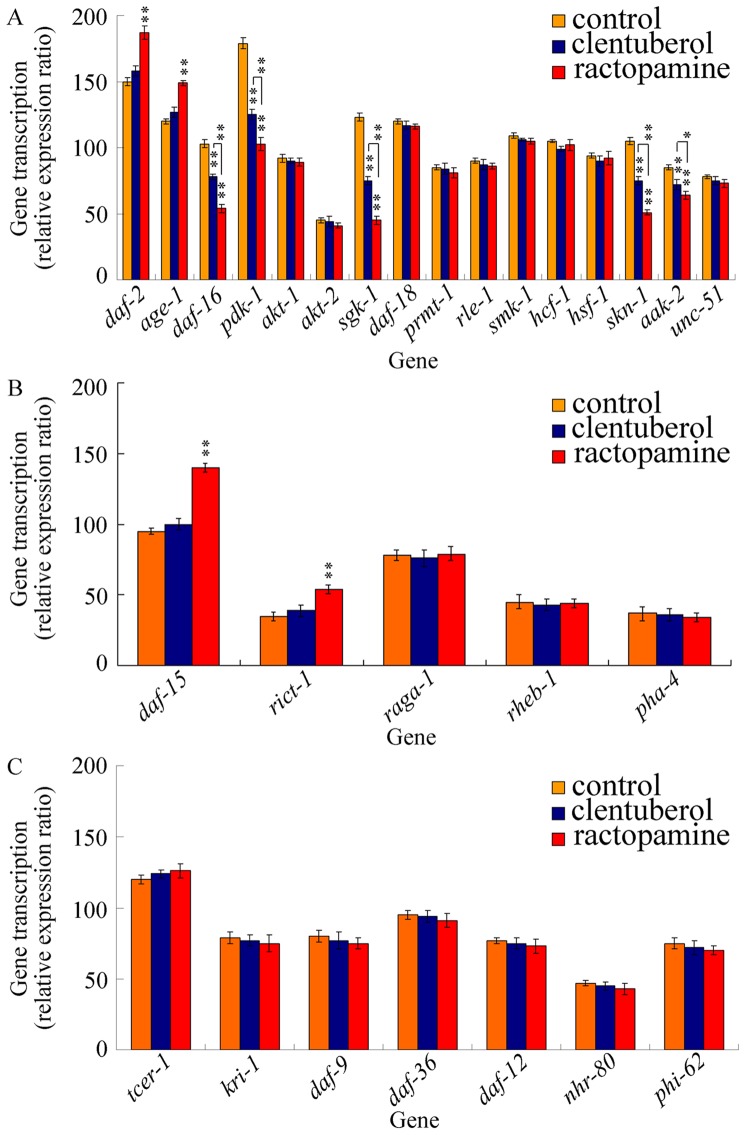
Effects of clenbuterol or ractopamine exposure on expression patterns of genes required for aging control. (A) Effects of clenbuterol or ractopamine exposure on expression patterns of genes in insulin/IGF-1 signaling pathway. (B) Effects of clenbuterol or ractopamine exposure on expression patterns of genes in TOR signaling pathway. (C) Effects of clenbuterol or ractopamine exposure on expression patterns of genes in germline signaling pathway. Exposures were performed from L1-larvae to adult (prolonged exposure) at the concentration of 10 µg/L. Bars represent mean ± S.E.M. **P<0.01.

In *C. elegans*, the aging process is under the control of three major endocrine- and nutrient-sensing signaling pathways, the insulin/insulin-like growth factor (IGF), target of rapamycin (TOR), and germline signaling pathways [56]. To determine the molecular mechanism for clentuberol or ractopamine to reduce the lifespan of nematodes, we investigated the expression patterns of genes involved in these three signaling pathways in clentuberol or ractopamine exposed nematodes. The insulin/IGF signaling pathway includes *daf-2*, *age-1*, *daf-16*, *pdk-1*, *akt-1*, *akt-2*, *sgk-1*, *daf-18*, *prmt-1*, *rle-1*, *smk-1*, *hcf-1*, *hsf-1*, *skn-1*, *akk-2*, and *unc-51* genes (56; Table S1). After prolonged exposure, we found that 10 µg/L of clentuberol decreased expression levels of *daf-16*, *sgk-1*, *skn-1*, and *aak-2* genes (Fig. 6A). In contrast, after prolonged exposure, 10 µg/L of ractopamine not only decreased expression levels of *daf-16*, *sgk-1*, *skn-1*, and *aak-2* genes, but also increased expression levels of *daf-2* and *age-1* genes (Fig. 6A). More interestingly, ractopamine more severely decreased the expression levels of *daf-16*, *sgk-1*, *skn-1*, and *aak-2* genes than clentuberol (Fig. 6A). TOR signaling pathway includes *daf-15*, *rict-1*, *raga-1*, *rheb-1*, and *pha-4* genes (56; Table S1). After prolonged expression, clentuberol did not significantly affect TOR signaling; however, ractopamine increased expression levels of *daf-15* and *rict-1* genes (Fig. 6B). Germline signaling pathway includes *tcer-1*, *kri-1*, *daf-9*, *daf-36*, *daf-12*, *nhr-80*, and *phi-62* genes (56; Table S1). After prolonged expression, both clentuberol and ractopamine did not significantly influence the expression levels of genes in germline signaling pathway (Fig. 6C). Therefore, clentuberol and ractopamine may affect the longevity through different molecular mechanisms in nematodes.

**Figure 7:**
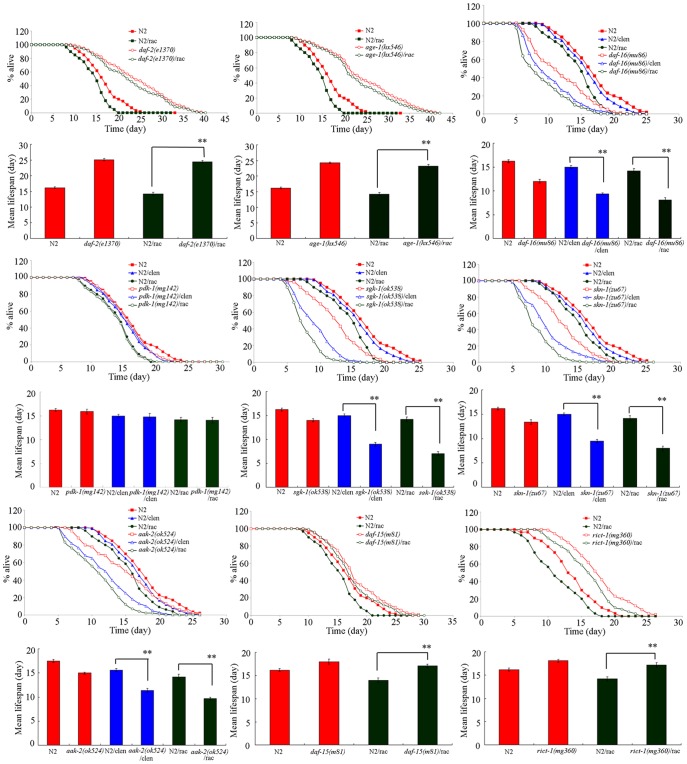
Lifespans in wild-type and mutants exposed to clenbuterol or ractopamine. Exposures were performed from L1-larvae to adult (prolonged exposure) at the concentration of 10 µg/L. Thirty nematodes were examined per treatment. clen, clenbuterol; rac, ractopamine. Bars represent mean ± S.E.M. **P<0.01.

To further confirm the functions of the dysregulated genes in regulating the toxicity formation from clentuberol or ractopamine, we used the corresponding mutants to investigate the lifespans of these mutants exposed to clentuberol or ractopamine. With the aid of lifespan as the endpoint, interestingly, we found that the *daf-16(mu86)*, *sgk-1(ok538)*, *skn-1(zu67)*, and *aak-2(ok524)* mutants had the susceptible property to the toxicity of clentuberol or ractopamine (Fig. 7). In contrast, the *daf-2(e1370)*, *age-1(hx546)*, *daf-15(m81)*, and *rict-1(mg360)* mutants had the resistant property to the toxicity of ractopamine (Fig. 7). These data further confirm the involvement of the related signaling pathways in regulating the toxicity formation from clentuberol or ractopamine.

## Discussion

In the present study, we first provide a series of evidence to indicate the usefulness of *C. elegans* assay system in assessing the *in vivo* toxicity of weight loss agents, such as clentuberol or ractopamine. With the aid of brood size, head thrash, body bend, intestinal autofluorescence, and intestinal ROS production as the endpoints, we detected the toxicity from acute exposure to clentuberol or ractopamine (Figs. 2 and 3). With the aid of body length, brood size, head thrash, body bend, intestinal autofluorescence, intestinal ROS production, and lifespan as the endpoints, we further detected the toxicity from prolonged exposure to clentuberol or ractopamine (Figs. 1–4). Using head thrash and body bend as the endpoints, we could observe the potential toxicity from acute exposure to 0.1 mg/L of clentuberol or ractopamine, and the potential toxicity from prolonged exposure to 0.01 µg/L of clentuberol or ractopamine (Fig. 2). Therefore, *C. elegans* may be a relatively sensitive assay system for toxicity assessment of food additives. Especially, the prolonged exposure assay system may be more suitable for detecting the potential toxicity of long-term adverse effects of weight loss agents. For example, prolonged exposure to 1–10 µg/L of clentuberol or ractopamine significantly decreased the body length of nematodes (Fig. 1). In contrast, acute exposure to all the examined concentrations of clentuberol or ractopamine did not noticeably influence the body length of nematodes (Fig. 1). Nevertheless, we did not observe the induction of lethality in nematodes exposed to clentuberol or ractopamine at the examined concentrations (Fig. 1). The careful selection of suitable and sensitive endpoints for toxicity assessment of weight loss agents should be paid attention to, since the toxicity of illegal weight loss agents may be not so severe as heavy metals [21], [23], [25]–[30] or organic pesticides [33]–[34].

Based on our data, we indicate that ractopamine may somewhat have more severe toxicity than clentuberol in nematodes. With the aid of head thrash, body bend, intestinal autofluorescence, and intestinal ROS production as the endpoints, after acute exposure, we detected the toxicity differences between clentuberol and ractopamine at concentrations of 1 and/or 5 mg/L (Figs. 2 and 3). With the aid of head thrash, body bend, intestinal autofluorescence, intestinal ROS production, and lifespan as the endpoints, after prolonged exposure, we observed the toxicity differences between clentuberol and ractopamine at relatively high concentrations (Figs. 2–4). Considering the fact that the toxicity for toxicants in *C. elegans* may be similar to that observed in mammals [16], [19], our data here imply the possible potential more severe toxicity of ractopamine than clentuberol in mammals.

Oxidative stress is a key mechanism for toxicants to induce the damage on nematodes [16], [18]. Our data demonstrate that both clentuberol and ractopamine induced the significant intestinal ROS production (Fig. 3B). Moreover, with the aid of the transgenic strain of *Ex*(P*dpy-30-sod-2*), we found that overexpression of *sod-2* gene in all cells effectively prevented the potential toxicity from clentuberol or ractopamine on growth, reproduction, locomotion behavior, intestinal development and lifespan in nematodes (Fig. 5). Therefore, oxidative stress is an important mechanism for clentuberol and ractopamine to induce the damage on nematodes.

Moreover, we found that clentuberol and ractopamine also induce the toxicity on nematodes through other mechanisms. In *C. elegans*, our data demonstrate that clentuberol and ractopamine caused the reduction in lifespan of animals possibly through different molecular mechanisms. Clentuberol might reduce the lifespan of nematodes through influencing the insulin/IGF signaling pathway; however, ractopamine might reduce the lifespan of nematodes through affecting both the insulin/IGF signaling pathway and the TOR signaling pathway (Fig. 6). In *C. elegans*, insulin/IGF-1 receptor (DAF-2) activates its tyrosine kinase activity and initiates a cascade of phosphorylation events that activate several kinases: phosphatidiylinositol 3-kinase (PI3K/AGE-1), 3-phosphoinositide-dependent kinase 1 (PDK-1), and serine/threonine-protein kinase (SGK-1) [56]. Ultimately, SGK-1 phosphorylates and inactivates the FOXO transcription factor DAF-16 and thereby blocks the transcription of targeted genes [57]. The catalytic subunit of AMP-activated protein kinase, AAK-2, is necessary for the long lifespan of *daf-2* mutants, which explains the energy mechanism [58]. TOR exists in two complexes, TORC1 and TORC2, and TORC1 and TORC2 contain different coactivators, DAF-15/Raptor and RICT-1/Rictor [59]. We hypothesize here that the more reduction in lifespan in ractopamine exposed nematodes than in clentuberol exposed nematodes may at least due to the induction of more severe alteration in genes required for aging control and the changes of more signaling pathways. Our data suggest that ractopamine more severely decreased the expression levels of *daf-16*, *sgk-1*, *skn-1*, and *aak-2* genes than clentuberol (Fig. 6A). In addition, ractopamine not only decreased the expression levels of *daf-16*, *sgk-1*, *skn-1*, and *aak-2* genes like clentuberol, but also increased the expression levels of *daf-2* and *age-1* genes (Fig. 6A).

Previous studies further imply the possible crosstalk between insulin/IGF signaling pathway and oxidative stress in clentuberol or ractopamine exposed nematodes. The transcription factor Skinhead (SKN-1) regulates resistance to oxidative stress and expression of detoxification genes in response to reduced isulin/IGF-1 signaling [60]. That is, the activated insulin/IGF signaling may at least partially contribute to the induction or regulation of oxidative stress in clentuberol or ractopamine exposed nematodes.

In conclusion, we provide the evidence in the present study to demonstrate that the *C. elegans* assay system was useful for assessment of possible *in vivo* toxicity from weight loss agents such as clentuberol and ractopamine. Our data imply that ractopamine exposure might induce more severe toxicity than clentuberol exposure in nematodes. Besides the oxidative stress, our results demonstrate that both insulin/IGF-1 signaling pathway and TOR signaling pathway were involved in the regulation of toxicity induction from clentuberol or ractopamine in nematodes. Moreover, we hypothesize that clentuberol and ractopamine might induce the toxicity on nematodes through different molecular mechanisms. Our data here will be helpful for our further understanding the potential damage from illegal use of weight loss agents on health of human and animals and the future design of effective strategies against the adverse effects from weight loss agents.

## Materials and Methods

### Reagents and strain preparation

Clentuberol and ractopamine were obtained from Sigma-Aldrich (St. Louis, MO, USA), and their purities were 95% and 99.5%, respectively. Exposure concentrations for clentuberol or ractopamine were 0.01–5 mg/L (acute exposure) or 0.01–10 µg/L (prolonged exposure).

Nematodes used were wild-type N2, mutants of *daf-2(31370)*, *age-1(hx546)*, *daf-16(mu86)*, *pdk-1(mg142)*, *sgk-1(ok538)*, *skn-1(zu67)*, *aak-2(ok524)*, *daf-15(m81)*, and *rict-1(mg360)*, and transgenic strain of *Ex*(P*dpy-30-sod-2*), which over-expresses the *sod-2* gene in all cells. Nematodes were maintained on nematode growth medium (NGM) plates seeded with *Escherichia coli* OP50 at 20°C [15]. Gravid nematodes were washed off the plates into centrifuge tubes, and were lysed with a bleaching mixture (0.45 M NaOH, 2% HOCl). Age synchronous populations of L1-larvae or young adult nematodes were obtained by the collection [21]. Nematodes were washed with a modified K medium (50 mM NaCl, 30 mM KCl, 10 mM NaOAc, pH 5.5) [61]. Exposures were performed from young adult for 24-hr (acute exposure) or from L1-larvae to adult (prolonged exposure) in K medium of 12-well sterile tissue culture plates at 20°C incubator in the presence of food.

### Lethality and growth

Lethality was evaluated by the percentage of survival animals. Following exposure, inactive ones were scored under a dissecting microscopy and nematodes were judged to be dead if they did not respond to stimulus using a small, metal wire. Fifty nematodes were examined per treatment. Growth was assessed by the body length, which was determined by measuring the flat surface area of nematodes using the Image-Pro® Express software. Twenty nematodes were examined per treatment. Three replicates were performed.

### Brood size and locomotion behavior

Reproduction was assessed by the brood size of adult nematodes. To assess brood size, we counted the number of offspring at all stages. Nematodes were transferred daily to new agar plates, until the completion of the egg laying period. Hatched progeny were allowed to grow to L1/L2 stage and counted manually. Twenty nematodes were examined per treatment. Three replicates were performed.

Locomotion behaviors of nematodes were evaluated by head thrash and body bend [62]. To assay head thrash, every examined nematode was transferred into a microtiter well containing 60 µL of modified K medium on the top of agar, and head thrashes were counted for 1-min after a 1-min recovery period. A thrash was defined as a change in the direction of bending at the mid body. To assay body bend, nematodes were picked onto a second plate and scored for the number of body bends in an interval of 20 sec. A body bend was counted as a change in the direction of the part of the nematodes corresponding to the posterior bulb of the pharynx along the *y* axis, assuming that the nematode was traveling along the *x* axis. Fifty nematodes were examined per treatment. Three replicates were performed.

### Intestinal autofluorescence

Intestinal autofluorescence caused by lysosomal deposits of lipofuscin can accumulate over time in aging nematodes [63]–[64]. Images were collected for endogenous intestinal fluorescence using a 525-nm bandpass filter and without automatic gain control in order to preserve the relative intensity of different animal's fluorescence. Observations of fluorescence were recorded and color images subjected to a common exposure time were taken for the documentation of results with Magnafire® software (Olympus, Irving, TX, USA). Lipofuscin levels were measured using ImageJ Software (NIH Image) by determining average pixel intensity in each animal's intestine. Twenty nematodes were examined per treatment. Three replicates were performed.

### ROS production

To quantify whether the clenbuterol or ractopamine exposure activated the oxidative damage, ROS production was assayed. The examined nematodes were transferred to M9 buffer containing 1 µM of 5-(and-6)-chloromethyl- 2′, 7′-dichlorodihydrofluorescein diacetate, acetyl ester (CM-H2DCFDA) to pre-incubate for 3-h at 20°C, and then mounted on agar pads for examination with a laser scanning confocal microscope (Leica, TCS SP2, Bensheim, Germany) at 488 nm of excitation wavelength and 510 nm of emission filter. Relative fluorescence intensities of the intestines were semi-quantified as described previously [30]. The semiquantified ROS was expressed as relative fluorescent units (RFU). Twenty nematodes were examined per treatment. Three replicates were performed.

### Lifespan assay

Lifespan assay was performed basically as described [65]–[66]. In the test, the hermaphrodites were transferred daily for the first 4 days of adulthood. Nematodes were checked every day and would be scored as dead when they did not move even after repeated taps with a pick. Thirty nematodes were examined per treatment. For lifespan, graphs are representative of at least three trials.

### Quantitative real-time polymerase chain reaction (PCR)

Total RNA was extracted using RNeasy Mini Kit (Qiagen). Total nematode RNA (∼1 µg) was reverse-transcribed using a cDNA Synthesis kit (Bio-Rad Laboratories). Quantitative reverse transcription PCR (RT-PCR) was run at the optimized annealing temperature of 58°C. Relative quantification of the targeted genes in comparison to the reference *act-1* gene was determined, and the final results were expressed as the relative expression ratio (between targeted genes and reference gene). The designed primers for targeted genes and reference *act-1* gene were shown in Table S2.

### DNA construct and germline transformation

To construct plasmid of P*dpy-30-sod-2*, *dpy-30* gene promoter fragment (1907 bp, PstI/BamHI) was subcloned into the pPD95_75 vector, and the full length of *sod-2* cDNA was inserted into the site of SmaI/KpnI of the pPD95_75 vector behind P*dpy-30* fragment. Transgenic nematodes of *Ex*(P*dpy-30-sod-2*) were generated as described [67]. Plasmids were injected as a mix at 20 ng/µL using P*dop-1::rfp* as a transgenic marker.

### Statistical analysis

All data were expressed as means ± standard error of the mean (S.E.M.). Statistical analysis was performed using SPSS 12.0 (SPSS Inc., Chicago, IL, USA). Analysis of variance (ANOVA) followed by Tukey post-hoc test was used to determine the significance of differences between the groups. Probability levels of 0.05 and 0.01 were considered statistically significant. The lifespan data were statistically analyzed using a 2-tailed 2 sample *t*-test (Minitab Ltd., Coventry, UK).

## Supporting Information

Table S1 **Information for genes required for aging control in** ***C. elegans*** **.**
(DOC)

Table S2 **Primers used for quantitative real-time polymerase chain reaction (PCR).**
(DOC)

## References

[1] Chan TY (1999) Health hazards due to clenbuterol residues in food. J Toxicol Clin Toxicol 37: 517–519. 10465253 10.1081/clt-100102525

[2] Yen M, Ewald MB (2012) Toxicity of weight loss agents. J Med Toxicol 8: 145–152. 22351299 10.1007/s13181-012-0213-7 PMC3550246

[3] Badino P, Odore R, Pagliasso S, Schiavone A, Girardi C, et al (2008) Steroid and beta-adrenergic receptor modifications in target organs of broiler chickens fed with a diet containing beta2-adrenergic agents. Food Chem Toxicol 46: 2239–2243. 18407392 10.1016/j.fct.2008.02.025

[4] Chai J, Xu Q, Dai J, Liu R (2013) Investigation on potential enzyme toxicity of clenbuterol to trypsin. Spectrochim Acta A Mol Biomol Spectrosc doi: 10.1016/j.saa.2012.12.017 10.1016/j.saa.2012.12.017 23314212

[5] Yaeger MJ, Mullin K, Ensley SM, Ware WA, Slavin RE (2012) Myocardial toxicity in a group of greyhounds administered ractopamine. Vet Pathol 49: 569–573. 21997565 10.1177/0300985811424752

[6] Lust EB, Barthold C, Malesker MA, Wichman TO (2011) Human health hazards of veterinary medications: information for emergency departments. J Emerg Med 40: 198–207. 20045604 10.1016/j.jemermed.2009.09.026

[7] Mazzanti G, Di Sotto A, Daniele C, Battinelli L, Brambilla G, et al (2007) A pharmacodynamic study on clenbuterol-induced toxicity: beta1- and beta2-adrenoceptors involvement in guinea-pig tacchycardia in an *in vitro* model. Food Chem Toxicol 45: 1694–1699. 17449161 10.1016/j.fct.2007.03.002

[8] Hoffman RJ, Hoffman RS, Freyberg CL, Poppenga RH, Nelson LS (2001) Clenbuterol ingestion causing prolonged tachycardia, hypokalemia, and hypophosphatemia with confirmation by quantitative levels. J Toxicol Clin Toxicol 39: 339–344. 11527226 10.1081/clt-100105152

[9] Bilkoo P, Thomas J, Riddle CD, Kagaoan G (2007) Clenbuterol toxicity: an emerging epidemic. A case report and review. Conn Med 71: 89–91. 17393901

[10] Gojmerac T, Pleadin J, Zuric M, Mirko L, Stipica C (2002) Effects of repeated growth-promoting doses of clenbuterol on the hepatic function of female pigs. Vet Hum Toxicol 44: 269–271. 12361107

[11] Patiyal SN, Katoch SS (2005) Beta-adrenoceptor agonist clenbuterol down-regulates matrix metalloproteinase (MMP-9) and results in an impairment of collagen turnover in mice left ventricle. Jpn J Physiol 55: 165–172. 16079024 10.2170/jjphysiol.R2118

[12] Downie D, Delday MI, Maltin CA, Sneddon AA (2008) Clenbuterol increases muscle fiber size and GATA-2 protein in rat skeletal muscle in utero. Mol Reprod Dev 75: 785–794. 17948249 10.1002/mrd.20795

[13] Douillard A, Galbes O, Rossano B, Vernus B, Bonnieu A, et al (2011) Time course in calpain activity and autolysis in slow and fast skeletal muscle during clenbuterol treatment. Can J Physiol Pharmacol 89: 117–125. 21326343 10.1139/y10-114

[14] Douillard A, Galbes O, Begue G, Rossano B, Levin J, et al (2012) Calpastatin overexpression in the skeletal muscle of mice prevents clenbuterol-indcued muscle hypertrophy and phenotypic shift. Clin Exp Pharmacol Physiol 39: 364–372. 22300302 10.1111/j.1440-1681.2012.05677.x

[15] Brenner S (1974) The genetics of *Caenorhabditis elegans* . Genetics 77: 71–94. 4366476 10.1093/genetics/77.1.71 PMC1213120

[16] Leung MCK, Williams PL, Benedetto A, Au C, Helmcke KJ, et al (2008) *Caenorhabditis elegans*: an emerging model in biomedical and environmental toxicology. Toxicol Sci 106: 5–28. 18566021 10.1093/toxsci/kfn121 PMC2563142

[17] Zhao Y-L, Wang D-Y (2012) Formation and regulation of adaptive response in nematode *Caenorhabditis elegans* . Oxid Med Cell Longev 2012: 564093. 22997543 10.1155/2012/564093 PMC3446806

[18] Zhao Y-L, Wu Q-L, Li Y-P, Wang D-Y (2013) Translocation, transfer, and in vivo safety evaluation of engineered nanomaterials in the non-mammalian alternative toxicity assay model of nematode *Caenorhabditis elegans* . RSC Adv 3: 5741–5757.

[19] Sprando RL, Olejnik N, Cinar HN, Ferguson M (2009) A method to rank order water soluble compounds according to their toxicity using *Caenorhabditis elegans*, a Complex Object Parametric Analyzer and Sorteer, and axenic liquid media. Food Chem Toxicol 7: 722–728. 10.1016/j.fct.2009.01.007 19162123

[20] Avila D, Helmcke K, Aschner M (2012) The *Caenorhabditis elegans* model as a reliable tool in neurotoxicology. Human Exp Toxicol 31: 236–243. 10.1177/0960327110392084 PMC4917367 21148196

[21] Donkin SG, Dusenbery DB (1993) A soil toxicity test using the nematode *Caenorhabditis elegans* and an effective method of recovery. Arch Environ Contam Toxicol 25: 145–151.

[22] Wang D-Y, Yang P (2007) Silver exposure causes transferable defects of phenotypes and behaviors in nematode *Caenorhabditis elegans* . Environ BioIndic 2: 89–98.

[23] Hu Y-O, Wang Y, Ye B-P, Wang D-Y (2008) Phenotypic and behavioral defects induced by iron exposure can be transferred to progeny in *Caenorhabditis elegans* . Biomed Environ Sci 21: 467–473. 19263801 10.1016/S0895-3988(09)60004-0

[24] Xing X-J, Du M, Xu X-M, Rui Q, Wang D-Y (2009) Exposure to metals induces morphological and functional alteration of AFD neurons in nematode *Caenorhabditis elegans* . Environ Toxicol Pharmacol 28: 104–110. 21783989 10.1016/j.etap.2009.03.006

[25] Xing X-J, Wang D-Y (2009) The lethality toxicities induced by metal exposure during development in nematode *Caenorhabditis elegans* . Bull Environ Contam Toxicol 83: 530–536. 19588066 10.1007/s00128-009-9816-3

[26] Benedetto A, Au C, Avila DS, Milatovic D, Aschner M (2010) Extracellular dopamine potentiates Mn-induced oxidative stress, lifespan reduction, and dopaminergic neurodegeneration in a BLI-3-dependent manner in *Caenorhabditis elegans* . PLoS Genet 6: e1001084. 20865164 10.1371/journal.pgen.1001084 PMC2928785

[27] Zhang Y-F, Ye B-P, Wang D-Y (2010) Effects of metal exposure on associative learning behavior in nematode *Caenorhabditis elegans* . Arch Environ Contam Toxicol 59: 129–136. 20044747 10.1007/s00244-009-9456-y

[28] Jiang B, Ren C, Li Y, Lu Y, Li W, et al (2011) Sodium sulfite is a potential hypoxia inducer that mimics hypoxic stress in *Caenorhabditis elegans* . J Biol Inorg Chem 16: 267–274. 21057967 10.1007/s00775-010-0723-1

[29] Liu P-D, He K-W, Li Y-X, Wu Q-L, Yang P, et al (2012) Exposure to mercury causes formation of male-specific structural deficits by inducing oxidative damage in nematodes. Ecotoxicol Environ Safety 79: 90–100. 22209111 10.1016/j.ecoenv.2011.12.007

[30] Wu Q-L, Qu Y-Y, Li X, Wang D-Y (2012) Chromium exhibits adverse effects at environmental relevant concentrations in chronic toxicity assay system of nematode *Caenorhabditis elegans* . Chemosphere 87: 1281–1287. 22336735 10.1016/j.chemosphere.2012.01.035

[31] Yu Z, Zhang J, Yin D (2012) Toxic and recovery effects of copper on *Caenorhabditis elegans* by various food-borne and water-borne pathways. Chemosphere 87: 1361–1367. 22386928 10.1016/j.chemosphere.2012.02.029

[32] Li Y-H, Ye H-Y, Du M, Zhang Y-F, Ye B-P, et al (2009) Induction of chemotaxis to sodium chloride and diacetyl and thermotaxis defects by microcystin-LR exposure in nematode *Caenorhabditis elegans* . J Environ Sci 21: 971–979. 10.1016/s1001-0742(08)62370-0 19862965

[33] Ruan Q-L, Ju J-J, Li Y-H, Liu R, Pu Y-P, et al (2009) Evaluation of pesticide toxicities with differing mechanisms using *Caenorhabditis elegans* . J Toxicol Environ Health A 72: 746–751. 19492238 10.1080/15287390902841532

[34] Boyd WA, Smith MV, Kissling GE, Rice JR, Snyder DW, et al (2009) Application of a mathematical model to describe the effects of chlorpyrifos on *Caenorhabditis elegans* development. PLoS ONE 4: e7204. 19753116 10.1371/journal.pone.0007024 PMC2737145

[35] Leung MCK, Goldstone JV, Boyd WA, Freedman JH, Meyer JN (2010) *Caenorhabditis elegans* generates biologically relevant levels of genotoxic metabolites from aflatoxin B1 but not benzo[α]pyrene *in vivo* . Toxicol Sci 118: 444–453. 20864627 10.1093/toxsci/kfq295 PMC2984530

[36] Ju J-J, Ruan Q-L, Li X-B, Liu R, Li Y-H, et al (2013) Neurotoxicological evaluation of microcystin-LR exposure at environmental relevant concentrations on nematode *Caenorhabditis elegans* . Environ Sci Pollut Res 20: 1823–1830. 10.1007/s11356-012-1151-2 22956115

[37] Rui Q, Lu Q, Wang D-Y (2009) Administration of *Bushenkangshuai Tang* alleviates the UV irradiation- and oxidative stress-induced lifespan defects in nematode *Caenorhabditis elegans* . Front Med China 3: 76–90.

[38] Shashikumar S, Rajini PS (2011) α-Tocopherol ameliorates cypermethrin-induced toxicity and oxidative stress in the nematode *Caenorhabditis elegans* . Ind J Biochem Biophy 48: 191–196. 21793311

[39] Sangha JS, Sun X, Wally OSD, Zhang K, Ji X, et al (2012) Liuwei Dihuang (LWDH), a traditional Chinese medicinal formula, protects against β-amyloid toxicity in transgenic *Caenorhabditis elegans* . PLoS ONE 7: e43990. 22952840 10.1371/journal.pone.0043990 PMC3431378

[40] Li Y-P, Li Y-X, Wu Q-L, Ye H-Y, Sun L-M, et al (2013) High concentration of vitamin E decreases thermosensation and thermotaxis learning and the underlying mechanisms in nematode *Caenorhabditis elegans* . PLoS ONE 8: e71180. 23951104 10.1371/journal.pone.0071180 PMC3741368

[41] Pluskota A, Horzowski E, Bossinger E, von Mikecz A (2009) In *Caenorhabditis elegans* nanoparticle-bio-interactions become transparent: silica-nanoparticles induce reproductive senescence. PLoS ONE 4: e6622. 19672302 10.1371/journal.pone.0006622 PMC2719910

[42] Roh J, Sim SJ, Yi J, Park K, Chung KH, et al (2009) Ecotoxicity of silver nanoparticles on the soil nematode *Caenorhabditis elegans* using functional ecotoxicogenomics. Environ Sci Technol 43: 3933–3940. 19544910 10.1021/es803477u

[43] Yu S-H, Rui Q, Cai T, Wu Q-L, Li Y-X, et al (2011) Close association of intestinal autofluorescence with the formation of severe oxidative damage in intestine of nematodes chronically exposed to Al_2_O_3_-nanoparticle. Environ Toxicol Pharmacol 32: 233–241. 21843804 10.1016/j.etap.2011.05.008

[44] Li Y-X, Yu S-H, Wu Q-L, Tang M, Pu Y-P, et al (2012) Chronic Al_2_O_3_-nanoparticle exposure causes neurotoxic effects on locomotion behaviors by inducing severe ROS production and disruption of ROS defense mechanisms in nematode *Caenorhabditis elegans* . J Hazard Mater 219–220: 221–230. 10.1016/j.jhazmat.2012.03.083 22521136

[45] Li Y-X, Wang W, Wu Q-L, Li Y-P, Tang M, et al (2012) Molecular control of TiO_2_-NPs toxicity formation at predicted environmental relevant concentrations by Mn-SODs proteins. PLoS ONE 7: e44688. 22973466 10.1371/journal.pone.0044688 PMC3433426

[46] Wu Q-L, Li Y-P, Tang M, Wang D-Y (2012) Evaluation of environmental safety concentrations of DMSA coated Fe_2_O_3_-NPs using different assay systems in nematode *Caenorhabditis elegans* . PLoS ONE 7: e43729. 22912902 10.1371/journal.pone.0043729 PMC3422352

[47] Wu Q-L, Nouara A, Li Y-P, Zhang M, Wang W, et al (2013) Comparison of toxicities from three metal oxide nanoparticles at environmental relevant concentrations in nematode *Caenorhabditis elegans* . Chemosphere 90: 1123–1131. 23062833 10.1016/j.chemosphere.2012.09.019

[48] Nouara A, Wu Q-L, Li Y-X, Tang M, Wang H-F, et al (2013) Carboxylic acid functionalization prevents the translocation of multi-walled carbon nanotubes at predicted environmental relevant concentrations into targeted organs of nematode *Caenorhabditis elegans* . Nanoscale 5: 6088–6096. 23722228 10.1039/c3nr00847a

[49] Li Y-X, Yu S-H, Wu Q-L, Tang M, Wang D-Y (2013) Transmissions of serotonin, dopamine and glutamate are required for the formation of neurotoxicity from Al_2_O_3_-NPs in nematode *Caenorhabditis elegans* . Nanotoxicology 7: 1004–1013. 22548316 10.3109/17435390.2012.689884

[50] Zhao Y-L, Wu Q-L, Tang M, Wang D-Y (2013) The *in vivo* underlying mechanism for recovery response formation in nano-titanium dioxide exposed *Caenorhabditis elegans* after transfer to the normal condition. Nanomedicine: Nanotechnol Biol Med doi: 10.1016/j.nano.2013.07.004 10.1016/j.nano.2013.07.004 23891985

[51] Qu Y, Li W, Zhou Y, Liu X, Zhang L, et al (2011) Full assessment of fate and physiological behavior of quantum dots utilizing *Caenorhabditis elegans* as a model organism. Nano Lett 11: 3174–3183. 21721562 10.1021/nl201391e

[52] Wu Q-L, Yin L, Li X, Tang M, Wang D-Y (2013) Contributions of altered permeability of intestinal barrier and defecation behavior to toxicity formation from graphene oxide in nematode *Caenorhabditis elegans* . Nanoscale 5: 9934–9943. 23986404 10.1039/c3nr02084c

[53] Wu Q-L, He K-W, Liu P-D, Li Y-X, Wang D-Y (2011) Association of oxidative stress with the formation of reproductive toxicity from mercury exposure on hermaphrodite nematode *Caenorhabditis elegans* . Environ Toxicol Pharmacol 32: 175–184. 21843797 10.1016/j.etap.2011.04.009

[54] Wang D-Y, Liu P-D, Yang Y-C, Shen L-L (2010) Formation of combined Ca/Cd toxicity on lifespan of nematode *Caenorhabditis elegans* . Ecotoxicol Environ Safety 73: 1221–1230. 20580433 10.1016/j.ecoenv.2010.05.002

[55] Ye B-P, Rui Q, Wu Q-L, Wang D-Y (2010) Metallothioneins are required for formation of cross-adaptation response to neurobehavioral toxicity from lead and mercury exposure in nematodes. PLoS ONE 5: e14052. 21124968 10.1371/journal.pone.0014052 PMC2987793

[56] Lapierre LR, Hansen M (2012) Lessons from *C. elegans*: signaling pathways for longevity. Trends Endocrinol Metab 23: 637–644. 22939742 10.1016/j.tem.2012.07.007 PMC3502657

[57] Kenyon CJ (2010) The genetics of ageing. Nature 464: 504–512. 20336132 10.1038/nature08980

[58] Apfeld J, O'Conner G, McDonagh T, DiStefano PS, Curtis R (2004) The AMP-activated protein kinase AAK-2 links energy levels and insulin-like signals to lifespan in *C. elegans* . Genes Dev 18: 3004–3009. 15574588 10.1101/gad.1255404 PMC535911

[59] Zoucu R, O'Connor G, McDonagh T, DiStefano PS, Curtis R (2011) mTOR: from growth signal integration to cancer, diabetes and ageing. Nat Rev Mol Cell Biol 12: 21–35. 21157483 10.1038/nrm3025 PMC3390257

[60] Tullet JM, Hertweck M, An JH, Baker J, Hwang JY, et al (2008) Direct inhibition of the longevity-promoting factor SKN-1 by insulin-like signaling in *C. elegans* . Cell 132: 1025–1038. 18358814 10.1016/j.cell.2008.01.030 PMC2367249

[61] Williams PL, Dusenbery DB (1990) Aquatic toxicity testing using the nematode *Caenorhabditis elegans* . Environ Toxicol Chem 9: 1285–1290. 11345460

[62] Wang D-Y, Liu P-D, Xing X-J (2010) Pretreatment with mild UV irradiation increases the resistance of nematode *Caenorhabditis elegans* to toxicity on locomotion behavior from metal exposure. Environ Toxicol Pharmacol 29: 213–222. 21787605 10.1016/j.etap.2010.01.002

[63] Shen L-L, Du M, Lin X-F, Cai T, Wang D-Y (2010) Genes required for the functions of olfactory AWA neuron regulate the longevity of *Caenorhabditis elegans* in an insulin/IGF signaling-dependent fashion. Neurosci Bull 26: 91–103. 20332814 10.1007/s12264-010-0162-6 PMC5552603

[64] Shen L-L, Hu Y-O, Cai T, Lin X-F, Wang D-Y (2010) Regulation of longevity by genes required for the functions of AIY interneuron in nematode *Caenorhabditis elegans.* . Mech Ageing Dev 131: 732–738. 21055415 10.1016/j.mad.2010.10.005

[65] He K-W, Shen L-L, Zhou W-W, Wang D-Y (2009) Regulation of aging by *unc-13* and *sbt-1* is temperature dependent in *Caenorhabditis elegans* . Neurosci Bull 25: 335–342. 19927169 10.1007/s12264-009-6123-2 PMC5552505

[66] Wang D-Y, Cao M, Dinh J, Dong Y-Q (2013) Methods for creating mutations in *C. elegans* that extend lifespan. Methods Mol Biol 1048: 65–75. 23929098 10.1007/978-1-62703-556-9_6

[67] Mello C, Fire A (1995) DNA transformation. Methods Cell Biol 48: 451–482. 8531738

