# Supplementary material for: Adverse Effects from Clenbuterol and Ractopamine on Nematode Caenorhabditis elegans and the Underlying Mechanism
Source: PLoS One. 2014 Jan 21;9(1):e85482. doi: 10.1371/journal.pone.0085482 (PMC3897430; doi:10.1371/journal.pone.0085482)
Supplement: Table S1 — Information for genes required for aging control in C. elegans . (DOC) [file pone.0085482.s001.doc]

***Supplementary Table 1. Information for genes required for aging control in*** C. elegans

| Genes | Products of the genes |
| --- | --- |
| *age-1* | phosphoinositide 3-kinase (PI3K) p110 catalytic subunit |
| *daf-2* | a receptor tyrosine kinase/insulin/IGF receptor |
| *daf-16* | forkhead box O (FOXO) transcription factor |
| *daf-18* | a lipid phosphatase homologous to the human PTEN tumor suppressor |
| *pdk-1* | 3-phosphoinositide-dependent kinase 1 ortholog |
| *akt-1* | an ortholog of the serine/threonine kinase Akt/PKB |
| *akt-2* | a homolog of the serine/threonine kinase Akt/PKB |
| *sgk-1* | a serine/threonine protein kinase |
| *prmt-1* | SAM-dependent methyltransferases |
| *rle-1* | E3 ubiquitin ligase |
| *smk-1* | an orthologous to SMEK proteins |
| *hcf-1* | a transcriptional regulator associated with histone modification enzymes |
| *hsf-1* | heat-shock transcription factor |
| *skn-1* | a transcription factor |
| *aak-2* | catalytic alpha subunit of AMP-activated protein kinases |
| *unc-51* | a serine/threonine protein kinase |
| *daf-15* | an ortholog of RAPTOR |
| *rict-1* | a component of the target of rapamycin complex 2 (TORC2) |
| *raga-1* | ortholog of the ras-related GTPase RagA |
| *rheb-1* | GTPase orthologous to the mammalian Rheb and Rheb1 GTPases |
| *pha-4* | a FoxA transcription factor |
| *phi-62* | endonuclease |
| *daf-9* | a cytochrome P450 of the CYP2 subfamily |
| *daf-36* | catalytic subunit of Rieske-like oxygenases |
| *daf-12* | a member of the steroid hormone receptor superfamily |
| *nhr-80* | a nuclear hormone receptor |
| *kri-1* | an ankyrin repeat and FERM domain-containing protein |
| *tcer-1* | transcription elongation regulator 1 protein |
