# Supplementary material for: Adverse Effects from Clenbuterol and Ractopamine on Nematode Caenorhabditis elegans and the Underlying Mechanism
Source: PLoS One. 2014 Jan 21;9(1):e85482. doi: 10.1371/journal.pone.0085482 (PMC3897430; doi:10.1371/journal.pone.0085482)
Supplement: Table S2 — Primers used for quantitative real-time polymerase chain reaction (PCR). (DOC) [file pone.0085482.s002.doc]

**Supplementary Table 2.** Primers used for quantitative real-time polymerase chain reaction (PCR)

| Gene | Forward primer | Reverse primer |
| --- | --- | --- |
| *act-1* | CTGCAGATGTGTGACGACGAGGTT | CTGCAGGAAGCACTTGCGGTGAAC |
| *age-1* | ATGGAAACCGCCGAGTGT | ATTGGCAGTCGGTTCAGG |
| *daf-2* | ATGTGGCGTGAGAATGAA | AGCCGAACACGAACAACA |
| *daf-16* | CGTTTCCTTCGGATTTCA | ATTCCTTCCTGGCTTTGC |
| *daf-18* | ATCATCATCCGCCGAGTC | ACCGTTGAGTCCTCCATC |
| *pdk-1* | TTCAGAGCCGTCAACCAG | GCTCACTTGCTCGGCTTT |
| *akt-1* | GGACAACCGTTTCCTGAG | GACGAACTTCTGCCGACT |
| *akt-2* | ATCAGCCGTTACCAGAGC | AAGGTTCCTTGACCGAGA |
| *sgk-1* | AAGACTGTTGACTGGTGGTG | AGACGAAGTGGCTGGTTG |
| *prmt-1* | AATCGCCGACAACAACCT | TCCCGTTTTCGGTACTCA |
| *rle-1* | CTCGCCCTGCTCTGCTAT | CCAAACACGGGATGACCT |
| *smk-1* | AACGAGACGCAACCAACC | CTCACCCAGCCTCCCAAT |
| *hcf-1* | TTTGGAGGTGGAAACGAA | TTGAGAAACGACGAAGCT |
| *hsf-1* | GCTCAATGCGTCAACAAC | GATGATTCTGCCGTGGTG |
| *skn-1* | TTATGCCAATACTCACCG | TGTAGGCGTAGTTGGATG |
| *aak-2* | TCATCCGCCTCTACCAAG | CTCGCCTCCATACTCATC |
| *unc-51* | AAACCATCGCCGAACAAC | AGACTCATCGCCTCTTCC |
| *daf-15* | TGGATTGGGTCGTCTGTG | CCAACTGGTTTACGTGGC |
| *rict-1* | GTCCGAACCCATACCACT | GCTTCTCGTAGCCATCCT |
| *raga-1* | GGATGCCGATGAAGTTAT | ATCCCTGGCGATGTAGTT |
| *rheb-1* | ATGGGATGCGAAGTTTGT | GACGCTCCGTTGGTGATA |
| *pha-4* | ATGAACGCTCAGGACTATCT | GGTGGTGCCAGTGGTAAA |
| *phi-62* | ACATTCAAGCCGTCACTC | TGCCGTGTTGTATTTATTCT |
| *daf-9* | TGCGTCAGAAGTGGAAGG | GGGTAGGAAGTTGCGAAG |
| *daf-36* | CACAAATCGGCTCCACCT | AACATCGCCAAGTCTCCT |
| *daf-12* | CAGCCACATTTATCACCG | GATTGTTAGTGGGCGTTG |
| *nhr-80* | ATCTCATCGTCCACCTCC | TATCCGTCTCATCCTCCC |
| *kri-1* | GTTCCTGGACTTTCACTT | ACATTGCTACGGACACTA |
| *tcer-1* | TCGACTCGTCCCATTAGC | GAAGCATTTGGCGAGGAC |
